# Doctor and practice characteristics associated with differences in patient evaluations of general practice

**DOI:** 10.1186/1472-6963-7-46

**Published:** 2007-04-03

**Authors:** Hanne N Heje, Peter Vedsted, Ineta Sokolowski, Frede Olesen

**Affiliations:** 1 The Department and Research Unit for General Practice, The University of Aarhus, Aarhus, Denmark; 2 The Research Unit for General Practice, The University of Aarhus, Aarhus, Denmark

## Abstract

**Background:**

Variation in patients' evaluation due to general practitioner (GP) and practice factors may provide information useful in a quality improvement context. However, the extent to which differences in patients' evaluation of the GPs are associated with differences in GP and practice characteristics must also be ascertained in order to facilitate comparison of adjusted patient evaluations between GPs. The aim of this study was to determine such associations in a setting where GPs serve a list of patients and act as gatekeepers.

**Methods:**

We carried out a patient evaluation survey among voluntarily participating GPs using the EUROPEP questionnaire, which produced 28,260 patient evaluations (response rate 77.3%) of 365 GPs. In our analyses we compared the prevalence of positive evaluations in groups of GPs.

**Results:**

Our principal finding was a negative association between the GP's age and the evaluation of all aspects, except accessibility. We also found an association between the way the practice was organised and the patients' evaluation of accessibility, with GPs in single-handed practices getting far the most positive evaluations. Long weekly working hours were associated with more positive evaluations of all dimensions except accessibility, whereas more than 0.5 full-time employees per GP, a higher number of listed patients per GP and working in a training practice were associated with negative evaluation of accessibility.

**Conclusion:**

GP characteristics are mainly associated with patients' experience of interpersonal aspects of care, while practice characteristics are associated with evaluation of accessibility. These differences need to be accounted for when comparing patient evaluations of different practices.

## Background

Ongoing efforts to improve the quality of general practice care increasingly include patients' evaluation of various aspects of care [1-3]. Variation in patient evaluation of general practice reflects differences in performance, which, to some extent, may be associated with GP and practice characteristics, and differences in the patients' perception of performance. Crude unadjusted results, though, may serve best for quality improvement at a practice level as they reflect the extent to which the GPs succeed in meeting the patients' individual needs and dealing with different working conditions [4].

When comparing practices and when holding practices against standards comparability is obtained through standardisation – that is adjustment for systematic variation in patient, GP and practice characteristics between the compared groups. Studies of associations between patient evaluations and patient, GP and practice characteristics are needed to determine which characteristics to adjust for. Knowledge of such associations may also serve quality improvement purposes at regional and national levels.

Earlier studies report a positive association between patients' evaluation and the GP's age [5], but a negative association between the evaluation and the GP's age and seniority has also been found [6,7]. Baker [5,6] and Campbell [8] found an inverse relation between assessment and total list size as well as list size per GP. Lin [9] found better evaluations of group practices than of single-handed practices, while Hombergh [10] found the opposite, which was also supported by Wensing [11], who found better ratings of practices with fewer and full-time working GPs. This supports the general finding that patients prefer personal, continuous care [10,12,13]. In addition, Baker [5,6] found that being a training practice produced less positive evaluations.

These results derive from studies in different settings and with different degrees of adjustment for differences in patient characteristics. Hence, there seems to be a need for a sufficiently powered comprehensive study of associations between patient evaluations and GP and practice characteristics adjusted for systematic variation in patient characteristics. Such a study will provide us with information about GP and practice factors of significance to the quality of general practice and information on how to standardize patient evaluation results for comparison between (groups of) practices.

On this background the aim of the present study is to determine to which extent variation in patient evaluations of the GPs are associated with GP and practice characteristics.

## Methods

### Study population

This study was carried out in a general practice setting where self-employed GPs work as gatekeepers for the public health services on a contract basis serving patients on their list (a brief introduction to the Danish general practice is given in Additional file 1). During 2002–4 all 2181 GPs from ten Danish counties received an invitation to carry out patient evaluations of their practices. A total of 365 GPs (16%–34% of all GPs in these counties) entered the project and undertook an evaluation. This was part of the national project on patient evaluations, the DanPEP. The participating GPs handed out questionnaires to 100 successive patients seen by the GP in the surgery or at home visits. The patients were at least 18 years of age, were listed in the practice and accepted a Danish language questionnaire. They were informed that their replies were anonymous to the doctor. Each questionnaire was identified by a serial number connecting it with the GP who handed it out and with the patient. All questionnaires not distributed within two weeks could be returned to the research office. All GPs filled in a form with information about the GP and the practice. Practice information from GPs working in the same practice was cross-checked.

### The questionnaire

The questionnaire contained the 23 items forming the EUROPEP instrument, which is a European validated instrument for patient evaluation of general practice care based on literature analysis and surveys of patients' expectations and opinions on good care [14,15]. The 23 items are displayed in Additional file 2.

These questions covered specific aspects of general practice and were grouped into five dimensions: doctor-patient relationship, medical care, information and support, organisation of care and accessibility. The answers were marked on a 5-point scale ranging from "poor" to "excellent", with "acceptable" as the middle value. Alternatively, the patients could choose a sixth category "not able to answer/not relevant". The questionnaire also included questions about the patient's gender, age, educational level, frequency of attendance to a general practice, time listed with the GP, self-rated health [16] and chronic conditions.

The patients were asked to assess the GP they considered to be their personal GP (and his practice when assessing aspects of accessibility) based on their contact experience over the past 12 months. They were also asked to write the GP's name on the questionnaire to confirm which GP was assessed and to allow individual assessment of GPs in partnership practices. The questionnaires were returned by the patients in prepaid envelopes to the research office.

In order to be able to carry out the reminder procedure, the GPs recorded the names, addresses and serial numbers from the questionnaires handed out. Reminders with new questionnaires were sent out by the research office to non-responding patients three to five weeks after the GPs' distributed the questionnaires and the patient lists were then destroyed. At the research office, the diagnoses reported by patients with chronic conditions were coded according to the major ICPC-2 groups [17].

### Assessments

For each dimension, a patient's evaluation was included in the calculations only if 50% or more of the items had been answered in one of the six categories. An answer was considered positive if it fell into one of the two most favourable categories. An assessment of a dimension was categorised as 100%, 50–99% or 0–49% positive depending on the proportion of positively evaluated items in that dimension. In the analyses we compared the prevalence of assessments in the 100% category between strata and the prevalence in the 0–49% category, respectively.

### General practitioners

The gender and age of the GPs, their seniority in present practice, number of weekly working hours (out-of-hours-duties, teaching away from practice and consultative services were not included), number of listed patients and full-time working staff per GP were categorised as displayed in Table 1 (in shared single-handed practices we only counted one GP). Practices were registered as urban, rural or mixed. They were divided into categories of single-handed, shared single-handed, groups of single-handed or partnership practices with doctors working either full-time or part-time (practice types are explained in Additional file 1). Involvement in education was registered as education given in the practice and/or in settings outside the practice or as no involvement.

**Table 1:** Distribution of characteristics among participating general practitioners.

		**N**	**%**
**Gender**			
N = 365	Female	136	37.3
	Male	229	62.7
**Age **(years)			
N = 365	30 – 39	21	5.8
	40 – 49	125	34.3
	50 – 59	195	53.4
	60 +	24	6.6
**Seniority in present practice **(years)			
N = 255	0 – 2	20	7.8
	3 – 5	53	20.8
	6 – 10	41	16.1
	11 – 20	86	33.7
	20 +	55	21.6
**Working hours **(hours/week)			
N = 360	0 – 21	12	3.3
	22 – 37	131	36.4
	38 – 44	124	34.4
	45 +	93	25.8
**Urbanity**			
N = 361	Urban	190	52.6
	Rural	44	12.2
	Urban/rural	127	35.2
**List Size **(patients/GP*)			
N = 360	0 – 1000	31	8.6
	1001 – 1600	217	60.3
	1601 – 2000	93	25.8
	2001 +	19	5.3
**Practice organisation**			
N = 364	Single-handed	80	22.0
	Shared single-handed	12	3.3
	Group of single-handed	36	9.9
	Partnership	165	45.3
	Part-time partnership	71	19.5
**Staff **(full-time employees/GP)			
N = 358	0 – 0.5	69	19.3
	0.51 – 0.86	131	36.6
	0.87 – 1	107	29.9
	1.01 – 1.49	37	10.3
	1.5 +	14	3.9
**Teaching out of practice**			
N = 359	No	270	75.2
	Yes	89	24.8
**Training practice**			
N = 358	No	120	33.5
	Yes	238	66.5

* General practitioner

### Analyses

Associations between the GP and practice characteristics and the assessment scores for each of the five dimensions were estimated as prevalence ratios (PR). The PRs with 95% confidence intervals (95% CI) were chosen instead of odds ratios, which would tend to overestimate the associations owing to the high prevalence of the variables [18,19].

In the first model we estimated the crude PRs between the GP and practice characteristics and the proportion of 100% and 0–49% positive evaluations of each dimension. Based on these univariate analyses we identified confounding GP and practice variables. Hence, we found statistically significant correlations between GP age, gender and seniority and between organisation of practice, list size per GP, weekly working hours, urbanity and staff per GP and a high collinearity between GPs' age and seniority. GP age was more closely associated with patient evaluation than seniority and was therefore chosen as the adjusting variable. List size and urbanity were not associated with assessment and, hence, despite correlation with other variables, did not act as confounders.

In the next model we estimated the association between the GP and practice characteristics and the evaluation adjusted for patient characteristics (patient-adjusted PR). The confounding patient variables (patients' age and gender, frequency of attending a GP and self-rated health) were identified from analyses of associations between patient characteristics and their GP evaluation performed on the data set also used in the present study (*Heje et al., submitted*).

In the last model we included the confounding GP and practice variables resulting from the analyses in the first model, which were GP gender, age and weekly working hours, organisation of practice and staff (number of employees converted into full-time) per GP, along with the patient variables used in the former model and calculated the fully adjusted PRs for the associations between GP and practice characteristics and the patients' evaluations.

#### Dependent variable

We did two sets of analyses. In the first set the dependent variable was the prevalence of assessments in the 100% category. In the second set it was the prevalence of assessments in the 0–49% category.

#### Independent variables

Depending of the model (crude, patient adjusted or fully adjusted), our independent variables were GP gender, age and weekly working hours, organisation of practice and staff per GP and patient age, gender, frequency of attending a GP and self-rated health.

#### Modelling data

We used generalised linear models (GLM) with log link for Bernoulli family, i.e. modelling the PRs. Due to the high prevalences, some of the adjusted GLM analyses could not converge using the Bernoulli family. In these situations, we used the Poisson regression [20,21]. Model fit for each multivariate model was tested using the Hosmer-Lemeshow test for goodness of fit [22] developed for testing logistic modelling. However, where the Poisson regression was used, the high prevalences could produce estimated probabilities greater than one which would hamper the Hosmer-Lemeshow goodness of fit test very much. In such situations, we therefore used the post-estimation goodness of fit test for Poisson regression based on deviance statistics [23].

Patient clustering by GPs produced relatively high intra-class correlation coefficients (ranging from 0.034 to 0.134). We accounted for patient clustering by using robust standard errors in all analyses [24,25]. Analyses were performed using complete data only, i.e., the univariate and the GLM analyses were performed using the same set of data so that an increasing number of missings would not explain differences in associations when adjusting for more variables. We used Stata 9.1 for data processing [26].

## Results

A total of 36,561 questionnaires were distributed by the 365 GPs. After a reminder procedure and exclusion of responses from patients not indicating which GP they assessed or assessing non-participating GPs, we had 28,260 (77.3%) valid responses. Characteristics of the evaluated GPs and their practices are displayed in Table 1. Respondent characteristics are shown in Table 2. There were more than twice as many female as male respondents (Table 2), which reflects that women are more inclined than men to attend a GP [27] and to respond to questionnaires (*Heje et al., submitted*).

**Table 2:** Distribution of characteristics among participating patients.

		**N**	**%**
**Gender**			
N = 27313	Female	18985	69.5
	Male	8328	30.5
**Age **(years)			
N = 27379	18–24	1284	4.7
	25–29	2296	8.4
	30–34	3060	11.2
	35–39	2872	10.5
	40–44	2392	8.7
	45–49	1988	7.3
	50–54	2169	7.9
	55–59	2376	8.7
	60–64	2109	7.7
	65–69	1945	7.1
	70–74	1813	6.6
	75–79	1551	5.7
	80+	1524	5.6
**Frequency of attendance **(latest 12 months)			
N = 25860	0–1	2434	9.4
	2–3	7848	30.4
	4–5	6535	25.3
	6–7	3534	13.7
	8–9	1840	7.1
	10+	3669	14.2
**Self-rated health**			
N = 27087	Excellent	2350	8.7
	Very good	8121	30.0
	Good	10488	38.7
	Fair	5141	19.0
	Poor	987	3.6

GP gender was not associated with evaluation outcome when we adjusted for patient, GP and practice confounders. However, adjustment did not neutralize the negative association found with GP age for all dimensions except accessibility. GPs aged 30–39 years received the most positive evaluations, whereas there was no difference between the other age categories. There was no association with seniority, neither before nor after adjustment.

In the following the word "association" refers to the fully adjusted PR, i.e. the PR adjusted for confounding patient, GP and practice variables and for patent clustering.

For the first four dimensions ("GP-patient-relationship", "medical care", "information and support" and "organisation of care"), we found a positive association between the evaluation and GP working hours in excess of 45 hours a week (Tables 3, 4, 5, 6). Patients' evaluation of the organisation of practice (e.g. continuity) was positively associated with the GP's weekly working hours exceeding 37 hours. There was no association between weekly working hours and patients' assessment of accessibility (Table 7).

**Table 3:** Dimension 1: Crude and adjusted associations between patients' evaluation of aspects of the doctor-patient-relationship.

		Most positive assessments*					**Poor assessments^†^**				
		
		**Prev^‡ ^(%)**	**Crude PR^§^**	**Pt-adj. PR^||^**	**F-adj. PR^¶^**	F-adj. 95%CI**	**Prev^‡ ^(%)**	**Crude PR^§^**	**Pt-adj. PR^||^**	**Adj. PR^¶^**	F-adj. 95%CI**
**Gender**											
(N = 365)	Female	62.8	1	1^††^	1^††^		15.4	1	1	1	
	Male	64.9	1.03	1.00	1.00	0.96; 1.04	13.8	0.90	0.97	0.99	0.88; 1.12
*Goodness of fit (p)*						1.00					0.34
**Age **(years)											
(N = 365)	-39	72.4	1	1^††^	1^††^		8.7	1	1	1	
	40–49	64.4	*0.89*	*0.89*	*0.87*	*0.82; 0.93*	*14.6*	*1.68*	*1.68*	*1.73*	*1.35; 2.22*
	50–59	63.1	*0.87*	*0.86*	*0.84*	*0.79; 0.89*	*14.9*	*1.71*	*1.75*	*1.84*	*1.45; 2.35*
	60 +	64.2	*0.89*	*0.85*	*0.84*	*0.78; 0.91*	*13.8*	*1.59*	*1.72*	*1.75*	*1.29; 2.36*
*Goodness of fit (p)*						1.00					0.34
**Seniority**^‡‡ ^(years)											
(N = 255)	0–2	66.4	1	1	1^††^		13.5	1	1	1	
	3–5	66.1	1.00	1.00	1.02	0.94; 1.11	13.3	0.98	0.99	0.93	0.64; 1.34
	6–10	65.8	0.99	0.97	1.02	0.92; 1.12	13.8	1.02	1.05	0.97	0.65; 1.45
	11–20	65.6	0.99	0.98	1.03	0.94; 1.13	13.1	0.97	0.99	0.89	0.60; 1.33
	21 +	64.7	0.98	0.95	0.99	0.90; 1.10	13.8	1.02	1.08	1.03	0.69; 1.52
*Goodness of fit (p)*						1.00					*0.01*
**Working hours/week**											
(N = 360)	-21	57.8	1	1^††^	1^††^		18.1	1	1	1	
	22–37	61.4	1.06	1.07	1.06	0.93; 1.20	16.2	0.90	0.89	0.94	0.73; 1.21
	38–44	64.3	1.11	1.11	1.11	0.98; 1.27	14.5	0.90	0.81	0.84	0.65; 1.10
	45 +	68.1	*1.18*	*1.17*	*1.19*	*1.04; 1.36*	*11.5*	*0.63*	*0.65*	*0.66*	*0.49; 0.87*
*Goodness of fit (p)*						1.00					0.34
**Urbanity**											
(N = 361)	Urban	62.3	1	1^††^	1^††^		15.4	1	1	1	
	Rural	65.1	1.04	1.01	1.00	0.95; 1.06	14.3	0.93	0.97	0.99	0.82; 1.20
	Urban/rural	66.5	*1.07*	*1.04*	1.03	0.99; 1.07	12.8	*0.83*	*0.85*	*0.88*	*0.78; 0.99*
*Goodness of fit (p)*						1.00					0.47
**List size **(patients/GP)											
(N = 360)	-1000	63.6	1	1^††^	1^††^		14.7	1	1	1	
	1001–1600	64.4	1.01	1.00	0.97	0.90; 1.05	14.8	1.01	1.01	1.10	0.89; 1.36
	1601–2000	64.6	1.02	1.02	0.99	0.91; 1.07	12.8	0.87	0.86	0.95	0.74; 1.21
	2000 +	63.1	0.99	0.99	0.96	0.87; 1.06	13.2	0.90	0.89	0.96	0.71; 1.28
*Goodness of fit (p)*						1.00					0.06
**Practice organisation**^§§^											
(N = 364)	Single	65.4	1	1	1^††^		13.3	1	1	1	
	Shared single	65.3	1.00	1.00	1.11	0.99; 1.25	14.9	1.12	1.06	0.82	0.57; 1.16
	Group of singles	58.5	*0.90*	*0.93*	0.98	0.91; 1.06	18.3	*1.37*	*1.28*	1.07	0.85; 1.34
	Partnership	64.3	0.98	0.99	1.01	0.97; 1.06	14.1	1.06	1.02	0.94	0.80; 1.10
	Part-time partnership	64.8	0.99	0.99	1.05	0.99; 1.12	14.3	1.07	1.04	0.89	0.73; 1.08
*Goodness of fit (p)*						1.00					0.34
**Staff **(employees/GP)^||||^											
(N = 358)	0–0.5	61.9	1	1^††^	1^††^		15.8	1	1	1	
	0.51–0.86	63.7	1.03	1.02	1.01	0.95; 1.07	15.0	0.95	0.97	1.01	0.86; 1.19
	0.87–1	65.0	1.05	1.03	1.01	0.95; 1.07	13.2	*0.84*	0.86	0.94	0.79; 1.12
	1.01–1.49	66.2	1.07	1.06	1.04	0.97; 1.11	13.0	0.82	0.83	0.89	0.71; 1.12
	1.5 +	65.9	1.06	1.05	1.03	0.94; 1.13	13.6	0.86	0.88	0.97	0.69; 1.35
*Goodness of fit (p)*						1.00					0.34
**Teaching out of practice**											
(N = 359)	No	64.1	1	1^††^	1^††^		14.4	1	1	1	
	Yes	64.0	1.00	0.99	0.99	0.95; 1.04	14.4	1.00	1.01	1.02	0.88; 1.18
*Goodness of fit (p)*						1.00					0.32
**Training practice**											
(N = 358)	No	64.4	1	1^††^	1^††^		14.5	1	1	1	
	Yes	64.0	0.99	0.99	0.98	0.94; 1.02	14.3	0.98	0.98	1.01	0.88; 1.15
*Goodness of fit (p)*						1.00					0.37

Prevalence ratio (PR) for 100% positive assessments and for 0–49% positive assessments associated with different patient characteristics. Crude PRs are adjusted for patient clustering. Patient-adjusted PRs are adjusted for patients' gender, age, frequency of attending a general practitioner (GP) and self-rated health and patient clustering. Fully adjusted PRs are adjusted for GPs' gender, age, weekly working hours, organisation of practice and staff in addition to patients' gender, age, frequency of attending a GP, self-rated health and patient clustering. The goodness of model fitting is shown regarding the fully adjusted PRs (p-values).

* Patients who marked 100% of the answered questions in one of the two most positive answering categories.

^† ^Patients who marked less than 50% (0–49%) of the answered questions in one of the two most positive answering categories.

^‡ ^Prevalence

^§ ^Crude prevalence ratio is the unadjusted prevalence ratio adjusted for patient clustering

^|| ^Patient-adjusted prevalence ratio is the prevalence ratio adjusted for patients' gender, age, frequency of attending a GP and self-rated health and patient clustering.

^¶ ^Fully adjusted prevalence ratio is the prevalence ratio adjusted for GPs' gender, age, weekly working hours, organisation of practice and staff in addition to patients' gender, age, frequency of attending a GP, self-rated health and patient clustering.

** 95% confidence interval for the fully adjusted prevalence ratio.

^†† ^Poisson regression with robust variance

^‡‡ ^Seniority in present practice

^§§ ^"Single" = single-haned, "Shared single" = two ore more GPs sharing a single-handed practice, "Group of singles" = single-handed GPs sharing premises and staff, "Partnership" = GPs sharing patient-list, premises and staff, "Part-time partnership" = partnership practices with all GPs working part-time

^|||| ^Number converted to full-time working employees

**Table 4:** Dimension 2: Crude and adjusted associations between patients' evaluation of aspects of the medical care.

		Most positive assessments*					**Poor assessments^†^**				
		
		**Prev^‡ ^(%)**	**Crude PR^§^**	**Pt-adj. PR^||^**	**Adj. PR^¶^**	Adj. 95%CI**	**Prev^‡ ^(%)**	**Crude PR^§^**	**Pt-adj. PR^||^**	**Adj. PR^¶^**	Adj. 95%CI**
**Gender**											
(N = 365)	Female	60.4	1	1^††^	1^††^		20.3	1	1	1	
	Male	61.5	1.02	0.99	0.99	0.96; 1.02	19.0	0.94	0.98	1.01	0.92; 1.10
*Goodness of fit (p)*						1.00					*0.001*
**Age **(years)											
(N = 365)	-39	66.9	1	1^††^	1^††^		15.4	1	1	1	
	40–49	61.2	*0.92*	*0.91*	*0.91*	*0.86; 0.96*	19.8	*1.28*	*1.28*	*1.30*	*1.08; 1.58*
	50–59	60.5	*0.91*	*0.89*	*0.88*	*0.83; 0.93*	19.8	*1.28*	*1.32*	*1.37*	*1.13; 1.65*
	60 +	60.2	*0.90*	*0.86*	*0.88*	*0.80; 0.96*	18.4	1.20	1.28	1.25	0.95; 1.65
*Goodness of fit (p)*						1.00					*0.001*
**Seniority ‡‡ **(years)											
(N = 255)	0–2	64.3	1	1^††^	1^††^		17.6	1	1	1	
	3–5	62.2	0.97	0.96	0.98	0.90; 1.06	19.3	1.10	1.10	1.08	0.85; 1.37
	6–10	62.3	0.97	0.94	0.97	0.88; 1.06	18.6	1.05	1.11	1.09	0.84; 1.42
	11–20	62.3	0.97	0.95	0.98	0.90; 1.08	18.3	1.04	1.07	1.05	0.81; 1.37
	21 +	63.4	0.99	0.94	0.98	0.89; 1.08	17.2	0.98	1.05	1.06	0.80; 1.40
*Goodness of fit (p)*						1.00					0.07
**Working hours/week**											
(N = 360)	-21	54.2	1	1^††^	1^††^		24.4	1	1	1	
	22–37	59.6	1.10	1.10	1.08	0.95; 1.23	20.9	0.85	0.87	0.89	0.70; 1.14
	38–44	60.4	1.12	1.11	1.10	0.96; 1.26	19.8	0.81	0.84	0.87	0.67; 1.11
	45 +	64.6	*1.19*	*1.18*	*1.18*	*1.03; 1.35*	16.6	*0.68*	*0.70*	*0.72*	*0.56; 0.93*
*Goodness of fit (p)*						1.00					*0.001*
**Urbanity**											
(N = 361)	Urban	59.9	1	1^††^	1^††^		20.3	1	1	1	
	Rural	60.3	1.01	0.98	0.98	0.93; 1.04	20.6	1.01	0.97	1.04	0.91; 1.20
	Urban/rural	63.1	*1.05*	1.04	1.02	0.99; 1.06	17.9	*0.89*	*0.90*	0.91	0.83; 1.00
*Goodness of fit (p)*						1.00					*0.007*
**List size **(patients/GP)											
(N = 360)	-1000	59.6	1	1^††^	1^††^		22.1	1	1	1	
	1001–1600	60.9	1.02	1.01	0.98	0.90; 1.06	19.6	0.89	0.90	0.98	0.82; 1.18
	1601–2000	62.5	1.05	1.05	1.00	0.92; 1.09	18.0	*0.81*	*0.81*	0.93	0.76; 1.13
	2000 +	60.9	1.02	1.01	0.97	0.87; 1.07	19.7	0.89	0.90	1.02	0.81; 1.29
*Goodness of fit (p)*						1.00					*0.002*
**Practice organisation**^§§^											
(N = 364)	Single	61.7	1	1^††^	1^††^		17.8	1	1	1	
	Shared single	59.0	0.96	0.97	1.04	0.92; 1.18	22.8	1.28	1.23	1.04	0.79; 1.37
	Group of singles	57.8	0.94	0.96	1.01	0.94; 1.08	22.3	*1.26*	*1.21*	1.07	0.91; 1.25
	Partnership	61.7	1.00	1.01	1.03	0.98; 1.08	19.4	1.09	1.07	1.01	0.90; 1.14
	Part-time partnership	60.8	0.99	0.99	1.03	0.97; 1.09	19.7	1.11	1.11	1.01	0.86; 1.17
*Goodness of fit (p)*						1.00					*0.001*
**Staff **(employees/GP)^||||^											
(N = 358)	0–0.5	59.1	1	1^††^	1^††^		21.4	1	1	1	
	0.51–0.86	60.9	1.03	1.02	1.01	0.96; 1.06	19.7	0.92	0.93	0.97	0.86; 1.09
	0.87–1	61.8	1.04	1.03	1.01	0.96; 1.07	18.8	0.88	0.89	0.96	0.84; 1.09
	1.01–1.49	61.6	1.04	1.03	1.02	0.95; 1.08	17.9	*0.84*	*0.84*	0.89	0.76; 1.04
	1.5 +	66.1	*1.12*	*1.11*	*1.08*	*1.01; 1.15*	17.0	*0.80*	*0.81*	0.90	0.77; 1.05
*Goodness of fit (p)*						1.00					*0.001*
**Teaching out of practice**											
(N = 359)	No	61.3	1	1^††^	1^††^		19.2	1	1	1	
	Yes	60.4	0.99	0.99	0.99	0.95; 1.03	20.0	1.00	1.03	1.03	0.93; 1.14
*Goodness of fit (p)*						1.00					0.08
**Training practice**											
(N = 358)	No	61.8	1	1^††^	1^††^		19.1	1	1	1	
	Yes	60.8	0.98	0.99	0.97	0.94; 1.01	19.6	1.03	1.01	1.03	0.93; 1.13
*Goodness of fit (p)*						1.00					0.08

Prevalence ratio (PR) for 100% positive assessments and for 0–49% positive assessments associated with different patient characteristics. Crude PRs are adjusted for patient clustering. Patient-adjusted PRs are adjusted for patients' gender, age, frequency of attending a general practitioner (GP) and self-rated health and patient clustering. Fully adjusted PRs are adjusted for GPs' gender, age, weekly working hours, organisation of practice and staff in addition to patients' gender, age, frequency of attending a GP, self-rated health and patient clustering. The goodness of model fitting is shown regarding the fully adjusted PRs (p-values).

* Patients who marked 100% of the answered questions in one of the two most positive answering categories.

^† ^Patients who marked less than 50% (0–49%) of the answered questions in one of the two most positive answering categories.

^‡ ^Prevalence

^§ ^Crude prevalence ratio is the unadjusted prevalence ratio adjusted for patient clustering

^|| ^Patient-adjusted prevalence ratio is the prevalence ratio adjusted for patients' gender, age, frequency of attending a GP and self-rated health and patient clustering.

^¶ ^Fully adjusted prevalence ratio is the prevalence ratio adjusted for GPs' gender, age, weekly working hours, organisation of practice and staff in addition to patients' gender, age, frequency of attending a GP, self-rated health and patient clustering.

** 95% confidence interval for the fully adjusted prevalence ratio.

^†† ^Poisson regression with robust variance

^‡‡ ^Seniority in present practice

^§§ ^"Single" = single-haned, "Shared single" = two ore more GPs sharing a single-handed practice, "Group of singles" = single-handed GPs sharing premises and staff, "Partnership" = GPs sharing patient-list, premises and staff, "Part-time partnership" = partnership practices with all GPs working part-time

^|||| ^Number converted to full-time working employees

**Table 5:** Dimension 3: Crude and adjusted associations between patients' evaluation of aspects of the information and support.

		Most positive assessments*					**Poor assessments^†^**				
		
		**Prev^‡ ^(%)**	**Crude PR^§^**	**Pt-adj. PR^||^**	**Adj. PR^¶^**	Adj. 95%CI**	**Prev^‡ ^(%)**	**Crude PR^§^**	**Pt-adj. PR^||^**	**Adj. PR^¶^**	Adj. 95%CI**
**Gender**											
(N = 365)	Female	62.9	1	1^††^	1^††^		21.2	1	1	1	
	Male	65.2	1.04	1.01	1.01	0.97; 1.04	18.7	*0.88*	0.94	0.93	0.85; 1.02
*Goodness of fit (p)*						1.00					0.48
**Age **(years)											
(N = 365)	-39	71.9	1	1^††^	1^††^		12.7	1	1	1	
	40–49	64.2	*0.89*	*0.89*	*0.88*	*0.83; 0.93*	*19.7*	*1.55*	*1.55*	*1.59*	*1.28; 1.98*
	50–59	63.8	*0.89*	*0.87*	*0.86*	*0.81; 0.90*	*20.1*	*1.58*	*1.61*	*1.71*	*1.37; 2.12*
	60 +	64.2	*0.89*	*0.85*	*0.85*	*0.79; 0.93*	*20.2*	*1.59*	*1.73*	*1.78*	*1.35; 2.34*
*Goodness of fit (p)*						1.00					0.48
**Seniority**^‡‡ ^(years)											
(N = 255)	0–2	68.0	1	1^††^	1^††^		17.3	1	1	1	
	3–5	65.7	0.97	0.96	0.98	0.90; 1.06	18.3	1.06	1.07	1.01	0.76; 1.36
	6–10	66.3	0.97	0.95	0.98	0.90; 1.08	19.0	1.10	1.14	1.03	0.75; 1.40
	11–20	65.7	0.97	0.95	0.98	0.89; 1.07	18.0	1.04	1.07	0.95	0.71; 1.28
	21 +	65.4	0.96	0.92	0.96	0.87; 1.05	18.5	1.07	1.15	1.02	0.75; 1.37
*Goodness of fit (p)*						1.00					0.93
**Working hours/week**											
(N = 360)	-21	57.2	1	1^††^	1^††^		25.7	1	1	1	
	22–37	62.3	1.09	1.10	1.08	0.95; 1.24	21.0	0.82	0.82	0.86	0.68; 1.09
	38–44	64.0	1.12	1.12	1.12	0.98; 1.28	19.7	*0.77*	*0.78*	0.80	0.62; 1.03
	45 +	68.3	*1.19*	*1.19*	*1.20*	*1.04; 1.37*	17.0	*0.66*	*0.69*	*0.70*	*0.54; 0.91*
*Goodness of fit (p)*						1.00					0.48
**Urbanity**											
(N = 361)	Urban	62.5	1	1^††^	1^††^		21.0	1	1	1	
	Rural	65.5	1.05	1.02	1.02	0.97; 1.07	19.5	0.93	0.97	0.99	0.85; 1.15
	Urban/rural	66.9	*1.07*	*1.05*	*1.04*	*1.01; 1.08*	17.5	*0.83*	*0.86*	*0.88*	*0.80; 0.97*
*Goodness of fit (p)*						1.00					*0.03*
**List size **(patients/GP)											
(N = 360)	-1000	63.5	1	1^††^	1^††^		20.2	1	1	1	
	1001–1600	64.3	1.01	1.01	0.96	0.89; 1.05	19.7	0.97	0.98	1.09	0.90; 1.31
	1601–2000	65.2	1.03	1.03	0.98	0.90; 1.07	18.7	0.92	0.92	1.04	0.85; 1.28
	2000 +	64.8	1.02	1.02	0.96	0.87; 1.07	19.3	0.95	0.95	1.08	0.84; 1.39
*Goodness of fit (p)*						1.00					0.29
**Practice organisation**^§§^											
(N = 364)	Single	65.8	1	1^††^	1^††^		18.3	1	1	1	
	Shared single	63.3	0.96	0.98	1.07	0.95; 1.19	19.9	1.08	1.04	0.82	0.61; 1.09
	Group of singles	60.8	*0.92*	0.95	1.01	0.94; 1.07	23.0	*1.25*	1.18	1.00	0.83; 1.19
	Partnership	64.4	0.98	0.99	1.01	0.97; 1.05	19.7	1.08	1.05	1.00	0.88; 1.13
	Part-time partnership	64.5	0.98	0.98	1.03	0.98; 1.09	19.2	1.05	1.03	0.91	0.77; 1.06
*Goodness of fit (p)*					1.00					0.48	
**Staff **(employees/GP)^||||^											
(N = 358)	0–0.5	62.1	1	1^††^	1^††^		21.8	1	1	1	
	0.51–0.86	64.2	1.03	1.03	1.02	0.97; 1.07	19.7	0.90	0.92	0.93	0.82; 1.05
	0.87–1	65.2	1.05	1.03	1.01	0.96; 1.07	18.9	0.87	0.90	0.93	0.81; 1.07
	1.01–1.49	65.6	1.06	1.05	1.03	0.97; 1.10	18.0	*0.82*	*0.83*	0.85	0.71; 1.01
	1.5 +	68.1	*1.10*	1.09	1.06	0.98; 1.14	17.3	*0.79*	*0.81*	0.86	0.71; 1.03
*Goodness of fit (p)*					1.00					0.48	
**Teaching out of practice**											
(N = 359)	No	64.5	1	1^††^	1^††^		19.7	1	1	1	
	Yes	64.0	0.99	0.99	0.99	0.95; 1.03	19.2	1.00	0.98	0.99	0.89; 1.10
*Goodness of fit (p)*						1.00					0.23
**Training practice**											
(N = 358)	No	64.7	1	1^††^	1^††^		19.5	1	1	1	
	Yes	64.3	0.99	1.00	0.99	0.95; 1.02	19.5	1.00	1.00	1.03	0.94; 1.14
*Goodness of fit (p)*						1.00					0.30

Prevalence ratio (PR) for 100% positive assessments and for 0–49% positive assessments associated with different patient characteristics. Crude PRs are adjusted for patient clustering. Patient-adjusted PRs are adjusted for patients' gender, age, frequency of attending a general practitioner (GP) and self-rated health and patient clustering. Fully adjusted PRs are adjusted for GPs' gender, age, weekly working hours, organisation of practice and staff in addition to patients' gender, age, frequency of attending a GP, self-rated health and patient clustering. The goodness of model fitting is shown regarding the fully adjusted PRs (p-values).

* Patients who marked 100% of the answered questions in one of the two most positive answering categories.

^† ^Patients who marked less than 50% (0–49%) of the answered questions in one of the two most positive answering categories.

^‡ ^Prevalence

^§ ^Crude prevalence ratio is the unadjusted prevalence ratio adjusted for patient clustering

^|| ^Patient-adjusted prevalence ratio is the prevalence ratio adjusted for patients' gender, age, frequency of attending a GP and self-rated health and patient clustering.

^¶ ^Fully adjusted prevalence ratio is the prevalence ratio adjusted for GPs' gender, age, weekly working hours, organisation of practice and staff in addition to patients' gender, age, frequency of attending a GP, self-rated health and patient clustering.

** 95% confidence interval for the fully adjusted prevalence ratio.

^†† ^Poisson regression with robust variance

^‡‡ ^Seniority in present practice

^§§ ^"Single" = single-haned, "Shared single" = two ore more GPs sharing a single-handed practice, "Group of singles" = single-handed GPs sharing premises and staff, "Partnership" = GPs sharing patient-list, premises and staff, "Part-time partnership" = partnership practices with all GPs working part-time

^|||| ^Number converted to full-time working employees

**Table 6:** Dimension 4: Crude and adjusted associations between patients' evaluation of aspects of the organisation of care.

		Most positive assessments*					**Poor assessments^†^**				
		
		**Prev^‡ ^(%)**	**Crude PR^§^**	**Pt-adj. PR^||^**	**Adj. PR^¶^**	Adj. 95%CI**	**Prev^‡ ^(%)**	**Crude PR^§^**	**Pt-adj. PR^||^**	**Adj. PR^¶^**	Adj. 95%CI**
**Gender**											
(N = 365)	Female	63.8	1	1^††^	1^††^		23.8	1	1	1	
	Male	66,6	*1,04*	1,00	1,00	0,97; 1,04	21,4	*0,90*	0,99	1,00	0,91; 1,10
*Goodness of fit (p)*						1.00					0.17
**Age **(years)											
(N = 365)	-39	71.2	1	1^††^	1^††^		16.8	1	1	1	
	40–49	65,5	*0,92*	*0,92*	*0,91*	*0,85; 0,97*	22,5	*1,34*	*1,35*	*1,40*	*1,10; 1,78*
	50–59	65,1	*0,91*	*0,90*	*0,88*	*0,83; 0,94*	22,6	*1,34*	*1,39*	*1,48*	*1,16; 1,87*
	60 +	66,1	0,93	*0,88*	*0,88*	*0,81; 0,97*	22,8	*1,36*	*1,50*	*1,54*	*1,14; 2,09*
*Goodness of fit (p)*						1.00					0.17
**Seniority**^‡‡ ^(years)											
(N = 255)	0–2	70.0	1	1^††^	1^††^		21.0	1	1	1	
	3–5	66,4	0,95	0,95	0,95	0,88; 1,03	22,1	1,05	1,05	1,02	0,79; 1,31
	6–10	67,0	0,96	0,94	0,95	0,87; 1,03	21,5	1,03	1,06	1,04	0,79; 1,35
	11–20	67,3	0,96	0,95	0,98	0,90; 1,07	20,5	0,98	1,00	0,90	0,70; 1,17
	21 +	68,2	0,97	0,94	0,98	0,89; 1,07	19,7	0,94	1,01	0,87	0,67; 1,14
*Goodness of fit (p)*						1.00					0.07
**Working hours/week**											
(N = 360)	-21	57.7	1	1^††^	1^††^		28.8	1	1	1	
	22–37	63,4	1,10	1,11	1,11	0,98; 1,26	24,4	0,85	*0,82*	0,85	0,68; 1,05
	38–44	65,8	*1,14*	*1,14*	*1,16*	*1,02; 1,32*	22,3	*0,78*	*0,77*	*0,77*	*0,61; 0,97*
	45 +	69,1	*1,20*	*1,19*	*1,22*	*1,07; 1,39*	18,7	*0,65*	*0,65*	*0,65*	*0,51; 0,83*
*Goodness of fit (p)*						1.00					0.17
**Urbanity**											
(N = 361)	Urban	64.1	1	1^††^	1^††^		23.6	1	1	1	
	Rural	66,1	1,03	1,00	1,00	0,95; 1,05	21,6	0,92	0,97	0,97	0,85; 1,11
	Urban/rural	68,0	*1,06*	*1,04*	1,03	0,99; 1,07	20,5	*0,87*	*0,90*	0,91	0,83; 1,01
*Goodness of fit (p)*						1.00					0.16
**List size **(patients/GP)											
(N = 360)	-1000	63.9	1	1^††^	1^††^		24.4	1	1	1	
	1001–1600	65,9	1,03	1,02	0,99	0,91; 1,07	22,5	0,92	0,93	1,01	0,84; 1,20
	1601–2000	65,9	1,03	1,04	1,00	0,92; 1,09	20,8	0,85	0,84	0,93	0,76; 1,14
	2000 +	66,4	1,04	1,04	0,99	0,89; 1,10	21,6	0,89	0,88	0,98	0,74; 1,30
*Goodness of fit (p)*						1.00					0.06
**Practice organisation**^§§^											
(N = 364)	Single	66.8	1	1^††^	1^††^		20.3	1	1	1	
	Shared single	64,6	0,97	0,99	1,07	0,96; 1,20	23,5	1,16	1,11	0,91	0,71; 1,17
	Group of singles	62,3	0,93	0,96	1,01	0,95; 1,08	25,3	*1,25*	1,17	1,02	0,84; 1,22
	Partnership	65,2	0,98	0,99	1,01	0,97; 1,05	22,9	1,13	1,09	1,03	0,91; 1,18
	Part-time partnership	67,0	1,00	1,01	*1,06*	*1,01; 1,12*	21,6	1,07	1,03	0,92	0,78; 1,08
*Goodness of fit (p)*						1.00					0.17
**Staff **(employees/GP)^||||^											
(N = 358)	0–0.5	63.8	1	1^††^	1^††^		23.9	1	1	1	
	0.51–0.86	65,3	1,02	1,02	1,01	0,96; 1,06	22,6	0,94	0,96	0,98	0,86; 1,11
	0.87–1	66,5	1,04	1,02	1,01	0,96; 1,06	21,4	0,89	0,93	0,98	0,85; 1,12
	1.01–1.49	66,5	1,04	1,03	1,02	0,96; 1,09	21,3	0,89	0,90	0,94	0,79; 1,12
	1.5 +	68,7	1,08	1,06	1,05	0,98; 1,13	20,2	0,84	0,86	0,94	0,77; 1,15
*Goodness of fit (p)*						1.00					0.17
**Teaching out of practice**											
(N = 359)	No	65.9	1	1	1^††^		22.0	1	1	1	
	Yes	64,5	0,98	0,98	0,98	0,94; 1,01	23,2	1,00	1,06	1,06	0,96; 1,17
*Goodness of fit (p)*						1.00					0.19
**Training practice**											
(N = 358)	No	66.2	1	1^††^	1^††^		21.6	1	1	1	
	Yes	65.3	0,99	0,99	0,98	0,94; 1,01	22.6	1,05	1,04	1,06	0,95; 1,17
*Goodness of fit (p)*						1.00					0.31

Prevalence ratio (PR) for 100% positive assessments and for 0–49% positive assessments associated with different patient characteristics. Crude PRs are adjusted for patient clustering. Patient-adjusted PRs are adjusted for patients' gender, age, frequency of attending a general practitioner (GP) and self-rated health and patient clustering. Fully adjusted PRs are adjusted for GPs' gender, age, weekly working hours, organisation of practice and staff in addition to patients' gender, age, frequency of attending a GP, self-rated health and patient clustering. The goodness of model fitting is shown regarding the fully adjusted PRs (p-values).

* Patients who marked 100% of the answered questions in one of the two most positive answering categories.

^† ^Patients who marked less than 50% (0–49%) of the answered questions in one of the two most positive answering categories.

^‡ ^Prevalence

^§ ^Crude prevalence ratio is the unadjusted prevalence ratio adjusted for patient clustering

^|| ^Patient-adjusted prevalence ratio is the prevalence ratio adjusted for patients' gender, age, frequency of attending a GP and self-rated health and patient clustering.

^¶ ^Fully adjusted prevalence ratio is the prevalence ratio adjusted for GPs' gender, age, weekly working hours, organisation of practice and staff in addition to patients' gender, age, frequency of attending a GP, self-rated health and patient clustering.

** 95% confidence interval for the fully adjusted prevalence ratio.

^†† ^Poisson regression with robust variance

^‡‡ ^Seniority in present practice

^§§ ^"Single" = single-haned, "Shared single" = two ore more GPs sharing a single-handed practice, "Group of singles" = single-handed GPs sharing premises and staff, "Partnership" = GPs sharing patient-list, premises and staff, "Part-time partnership" = partnership practices with all GPs working part-time

^|||| ^Number converted to full-time working employees

**Table 7:** Dimension 5: Crude and adjusted associations between patients' evaluation of aspects of the accessibility.

		Most positive assessments*					**Poor assessments^†^**				
		
		**Prev^‡ ^(%)**	**Crude PR^§^**	**Pt-adj. PR^||^**	**Adj. PR^¶^**	Adj. 95%CI**	**Prev^‡ ^(%)**	**Crude PR^§^**	**Pt-adj. PR^||^**	**Adj. PR^¶^**	Adj. 95%CI**
**Gender**											
(N = 365)	Female	24.6	1	1	1^††^		38.7	1	1	1	
	Male	33,1	*1,35*	*1,21*	1,09	0,98; 1,21	29,9	*0,77*	*0,83*	*0,91*	*0,84; 0,99*
*Goodness of fit (p)*						1.00					0.29
**Age **(years)											
(N = 365)	-39	25.8	1	1	1^††^		34.2	1	1	1	
	40–49	28,7	1,11	1,12	1,00	0,79; 1,26	34,8	1,02	1,03	1,09	0,91; 1,30
	50–59	30,5	1,18	1,15	0,99	0,78; 1,26	32,8	0,96	0,98	1,10	0,92; 1,32
	60 +	35,9	*1,39*	1,25	1,04	0,77; 1,39	26,2	0,77	0,84	0,98	0,77; 1,24
*Goodness of fit (p)*						1.00					0.29
**Seniority**^‡‡ ^(years)											
(N = 255)	0–2	25.1	1	1	1^††^		33.2	1	1	1	
	3–5	25,7	1,02	1,02	0,99	0,79; 1,24	37,3	1,12	1,14	1,16	0,95; 1,41
	6–10	32,5	1,29	1,24	1,05	0,81; 1,35	29,7	0,89	0,92	1,07	0,84; 1,36
	11–20	32,1	*1,28*	1,24	1,18	0,92; 1,51	31,6	0,95	0,97	1,08	0,85; 1,38
	21 +	34,6	*1,38*	*1,26*	1,26	0,96; 1,65	28,8	0,87	0,92	1,04	0,80; 1,35
*Goodness of fit (p)*						1.00					0.92
**Working hours/week**											
(N = 360)	-21	26.0	1	1	1^††^		31.4	1	1	1	
	22–37	27,6	1,06	1,11	1,14	0,83; 1,55	34,4	1,10	1,09	0,96	0,75; 1,24
	38–44	30,2	1,16	1,18	1,10	0,80; 1,50	34,1	1,09	1,10	1,02	0,79; 1,32
	45 +	33,3	1,28	1,27	1,00	0,72; 1,39	30,3	0,97	0,99	1,05	0,80; 1,37
*Goodness of fit (p)*						1.00					0.29
**Urbanity**											
(N = 361)	Urban	33.0	1	1	1^††^		30.1	1	1	1	
	Rural	30,0	0,91	*0,83*	0,97	0,84; 1,12	35,6	*1,18*	*0,97*	1,11	0,99; 1,25
	Urban/rural	25,5	*0,77*	*0,73*	0,91	0,82; 1,02	36,8	*1,22*	*1,26*	1,04	0,95; 1,14
*Goodness of fit (p)*						1.00					0.27
**List size **(patients/GP)											
(N = 360)	-1000	31.9	1	1	1^††^		27.4	1	1	1	
	1001–1600	29,0	0,91	0,89	0,89	0,74; 1,07	34,3	*1,25*	*1,25*	*1,27*	*1,05; 1,54*
	1601–2000	32,0	1,00	1,01	0,88	0,71; 1,08	31,6	1,15	1,13	*1,33*	*1,07; 1,64*
	2000 +	29,6	0,93	0,92	0,81	0,62; 1,04	33,8	1,23	1,21	*1,48*	*1,19; 1,83*
*Goodness of fit (p)*						1.00					0.74
**Practice organisation**^§§^											
(N = 364)	Single	46.2	1	1^††^	1^††^		18.7	1	1	1	
	Shared single	30,9	*0,67*	*0,70*	*0,56*	*0,44; 0,72*	29,5	*1,58*	*1,52*	*1,75*	*1,36; 2,25*
	Group of singles	33,8	*0,73*	*0,79*	*0,68*	*0,56; 0,82*	27,2	*1,46*	*1,39*	*1,53*	*1,23; 1,89*
	Partnership	24,6	*0,53*	*0,55*	*0,52*	*0,46; 0,58*	38,5	*2,06*	*2,01*	*2,07*	*1,76; 2,43*
	Part-time partnership	20,1	*0,44*	*0,45*	*0,40*	*0,34; 0,46*	42,0	*2,25*	*2,18*	*2,34*	*1,98; 2,78*
*Goodness of fit (p)*						1.00					0.29
**Staff **(employees/GP)^||||^											
(N = 358)	0–0.5	32.1	1	1	1^††^		31.1	1	1	1	
	0.51–0.86	27,4	*0,85*	*0,84*	*0,81*	*0,69; 0,94*	34,8	1,12	*1,15*	*1,14*	*1,00; 1,29*
	0.87–1	32,3	1,00	0,96	*0,76*	*0,64; 0,91*	31,7	1,02	1,06	*1,21*	*1,05; 1,39*
	1.01–1.49	26,0	0,81	*0,78*	*0,69*	*0,56; 0,85*	37,9	*1,22*	*1,24*	*1,27*	*1,08; 1,49*
	1.5 +	36,0	1,12	1,10	*0,73*	*0,57; 0,94*	25,3	0,81	0,84	1,21	0,98; 1,49
*Goodness of fit (p)*						1.00					0.29
**Teaching out of practice**											
(N = 359)	No	30.5	1	1	1^††^		33.1	1	1	1	
	Yes	28,2	0,92	0,92	0,92	0,83; 1,02	33.2	1,00	1,00	0,99	0,90; 1,08
*Goodness of fit (p)*						1.00					0.36
**Training practice**											
(N = 358)	No	37.2	1	1	1^††^		27,2	1	1	1	
	Yes	26.1	*0,70*	*0,71*	*0,88*	*0,80; 0,97*	36.2	*1,33*	*1,33*	1,07	0,97; 1,17
*Goodness of fit (p)*						1.00					0.17

Prevalence ratio (PR) for 100% positive assessments and for 0–49% positive assessments associated with different patient characteristics. Crude PRs are adjusted for patient clustering. Patient-adjusted PRs are adjusted for patients' gender, age, frequency of attending a general practitioner (GP) and self-rated health and patient clustering. Fully adjusted PRs are adjusted for GPs' gender, age, weekly working hours, organisation of practice and staff in addition to patients' gender, age, frequency of attending a GP, self-rated health and patient clustering. The goodness of model fitting is shown regarding the fully adjusted PRs (p-values).

* Patients who marked 100% of the answered questions in one of the two most positive answering categories.

^† ^Patients who marked less than 50% (0–49%) of the answered questions in one of the two most positive answering categories.

^‡ ^Prevalence

^§ ^Crude prevalence ratio is the unadjusted prevalence ratio adjusted for patient clustering

^|| ^Patient-adjusted prevalence ratio is the prevalence ratio adjusted for patients' gender, age, frequency of attending a GP and self-rated health and patient clustering.

^¶ ^Fully adjusted prevalence ratio is the prevalence ratio adjusted for GPs' gender, age, weekly working hours, organisation of practice and staff in addition to patients' gender, age, frequency of attending a GP, self-rated health and patient clustering.

** 95% confidence interval for the fully adjusted prevalence ratio.

^†† ^Poisson regression with robust variance

^‡‡ ^Seniority in present practice

^§§ ^"Single" = single-haned, "Shared single" = two ore more GPs sharing a single-handed practice, "Group of singles" = single-handed GPs sharing premises and staff, "Partnership" = GPs sharing patient-list, premises and staff, "Part-time partnership" = partnership practices with all GPs working part-time

^|||| ^Number converted to full-time working employees

We saw practically no association with practice urbanisation. The number of patients and the number of employees per GP were negatively associated with the experienced accessibility (Table 7), but not with the evaluation of the other dimensions (Tables 3, 4, 5, 6). The way the GPs had arranged themselves in practice played a minor role regarding the first four dimensions (Tables 3, 4, 5, 6), while there were major differences regarding accessibility. GPs in single-handed practices were assessed more positively than GPs in shared single-handed, groups of single-handed and partnership practices. Accessibility to GPs working part-time in partnership practices was assessed least favourably (Table 7).

Working in a training practice was only associated with the assessment of accessibility aspects (Table 7), where we found a negative association, while the evaluations were not associated with teaching outside the practice.

## Discussion

### Principal findings

We found a strong negative association between the GP's age and the evaluation of all aspects, except accessibility. We also found a strong association between the way the practice was organised and the patients' evaluation of accessibility, with GPs in single-handed practices getting far the most positive evaluations. Long weekly working hours were associated with more positive evaluations of all dimensions, except accessibility, whereas more than 0.5 full-time employees per GP, a comparatively high number of listed patients per GP and working in a training practice were associated with negative evaluation of accessibility.

### Discussion of methods

We chose to include the GPs' age as ten-year categories, and weekly working hours were categorised into two categories above and two categories below standard Danish full-time hours. For both variables we found general trends in their associations with assessments, which indicates that more narrow ranges would hardly have revealed additional patterns of associations. Seniority in present practice was preferred to seniority as a GP as the latter to some extent would correlate with the GP's age, while the former indicates the GP's opportunity to provide continuity.

We did not include locums and vocational trainees in the variable "number of GPs sharing premises", even though they may have influenced the total practice resources and the number of staff needed.

We chose a one-level model to account for patient clustering. However, GPs were also clustered in practices and a two-level model may therefore have been more applicable. Due to difficulties in converging the two-level model, we tested the differences between one- and two-level models and found that the variances between GPs were small, and differences in PR estimates were therefore negligible (below 2%).

Overall, the model fit showed to be good using the Bernoulli family and in the situations where the Poisson regression was used. As earlier explained in the methods section, the high prevalences forced us to use Poisson regression in some instances to enable the model to converge, which in this study did not change the estimates [20,21].

### Strengths

The study enjoyed a very high statistical precision, which meant that we were able to detect quite small statistically significant associations and one might question their clinical relevance. However, the precision was, indeed, considerable, so the risk of overlooking associations (type II-error) was accordingly extremely low.

In several cases, we found that GP and practice characteristics were differently associated with the evaluation of various aspects of care. Such differences may be blurred in measures of general satisfaction, which makes it difficult to compare our results with results from studies using this kind of measure [28,29].

### Weaknesses

The project was a part of a large national patient evaluation project. This may have introduced some methodological weaknesses in relation to the aim of this particular study. All GPs in the involved counties were invited, but those who entered the project were not necessarily a representative fraction. The method of patient inclusion should ideally secure a random draw from the attending part of the listed patients, well knowing that frequently attending patients would be overrepresented. We do not know to which extent GPs forgot to hand out questionnaires or more systematically left some patients out. We focused on adjusted associations between evaluations and GP and practice characteristics. Selection of GPs and patients therefore did not have the same impact as if the aim had been to investigate actual evaluation levels.

### Discussion of results

Our finding that GP gender was not associated with the evaluation is in agreement with earlier findings [6,7], whereas the statistically significantly more positive evaluation of the youngest GPs in this field represents new knowledge. Earlier studies have reported a positive association between patients' evaluation of general practice care and the GP's age [5], but negative associations between the evaluation and the GP's age and seniority have also been found [6,7]. We found no correlation between the GP's age and the number of patients per GP or practice organisation that could explain the association found. The negative association between GP age and the evaluation may indicate that patients experience younger GPs as more skilled or it may reflect possible deficiencies in the continuous medical education (CME) of older GPs. Maybe, patients expect more from older GPs or, maybe, GPs over time adjust their effort in order to counter burnout. We would have expected the impact of continuity in the relationship to be reflected in a positive association between GP age and the evaluation score. Maybe, a "shift of scenes" is sometimes appreciated by the patients. If the GP is not aware of this potential problem, continuity may imply a degree of preoccupancy that hinders him from perceiving and adjusting to changes in the patient's life situation and health. This challenges the perception of personal, continuous care as an unconditionally valuable quality [10,12,13]. Yet another possible explanation may be that the association originated in a cohort effect, reflecting improvement in the CME of GPs, including both technical and inter-personal competencies.

The GP's weekly working hours were not associated with the patients' perception of accessibility, but were positively associated with aspects of care organisation, including continuity, presumably because continuity is closely related to the GP personally, whereas lack of accessibility may be compensated for by the colleagues or managed otherwise.

The geographical location of the practice was not associated with the evaluation. It is surprising, though, to see that practising in rural districts was not negatively associated with the evaluation of accessibility, even though regions with shortage of GPs were included in this study. This may possibly be explained by an adjustment of patient expectations due to public discussion of the problem.

Like in earlier studies [5,6,8,30], the number of patients per GP was not consistently associated with the evaluation in the first four dimensions, but we were not surprised to see that it was negatively associated with the patients' perception of accessibility. Inversely, the number of staff was also negatively associated with accessibility. In Denmark, practice nurses and laboratory assistants can conduct consultations on their own if supervised by the GP. Delegating certain services to practice staff may induce a sense of limited access to the GP, which is supported by the fact that we found that GPs with more employees served larger lists of patients.

In concordance with earlier findings [8,31], there was a slight tendency towards more positive evaluation of the GP's technical care in part-time partnership practices, but otherwise practice organisation mainly influenced the patients' experience of accessibility. GPs in single-handed practices got, by far, the most positive assessments of all, while part-time partnership practices obtained the most negative assessments. This supports results from earlier studies [8,10,11]. More modest patient expectations of the single-handed GP's accessibility may explain this, or the explanation may be concealed by other factors regarding organisation, service and priorities that we have not been able to adjust for.

Baker [5,6] found training practices to obtain less favourable assessments than non-training practices. Being involved with teaching and training out of practice was not associated with the evaluation in our study and working in a training practice was only associated with a comparatively lower evaluation of accessibility.

### Policy and practice implications

Our results suggest adjustment for GP age whenever evaluation results are compared between practices. On the other hand, both the GPs themselves and policy makers must be aware of correctable consequences of increasing GP age. Depending on the situation, adjustment for practice organisation may also be appropriate, but as practice organisation may be closely interwoven with other organisational aspects, this may blur important differences between practices that ought to be addressed.

We found single-handed practice, a short patient list, only a few employees, and not being a training practice to be associated with a better patient-experienced accessibility to care. This is contradictory to the finding of better technical performance of GPs in larger partnerships [8,31], to the current trend in society that favours larger primary care units and that younger doctors wish to work part-time hours in partnership practices. Further studies are therefore needed to discover what lies behind these results and how practice may be organised in order to secure high professional standards and at the same time satisfy the patients' needs for personal continuity and accessibility. As this is in some way a general paradox concerning all practices, policy makers and GPs should come together to seek for possible solutions to this problem.

It cannot be concluded that patient evaluation results should always or never be adjusted for differences between practices. Adjustment may be appropriate when comparing results between (groups of) practices, but in the perspective of individual practices, one may claim that every GP must strive to satisfy his patients disregarding that his working conditions may be different from those of his colleagues.

## Conclusion

We found the GP's age to be negatively associated with the patients' evaluation of all aspects of care, except accessibility. We also found a strong association between the way the practice was organised and the patients' evaluation of accessibility, with GPs in single-handed practices getting far the most positive evaluations. We suggest that future evaluations be adjusted for differences in the GPs' age and practice organisation before comparing (groups of) practices. Long weekly working hours were associated with more positive evaluations of all dimensions, except accessibility, whereas more than 0.5 full-time employees per GP, a comparatively high number of listed patients per GP and working in a training practice were associated with negative evaluation of accessibility. These results may be used to adjust practice in order to increase the patient-experienced accessibility to care.

## Competing interests

The author(s) declare that they have no competing interests.

## Authors' contributions

HH, PV and FO planned the study, and HH carried out the patient evaluation survey assisted by the research secretariat. IS, PV and HH planned the statistical analyses, which were performed by IS. HH drafted the manuscript, which was rewritten by HH, PV, FO and IS. All authors read and approved the final manuscript.

## Pre-publication history

The pre-publication history for this paper can be accessed here:



## Supplementary Material

Additional File 1 Danish general practice. Click here for file

Additional File 2 The EUROPEP questionnaire. Click here for file
