# Supplementary material for: Doctor and practice characteristics associated with differences in patient evaluations of general practice
Source: BMC Health Serv Res. 2007 Apr 3;7:46. doi: 10.1186/1472-6963-7-46 (PMC1855053; doi:10.1186/1472-6963-7-46)
Supplement: Additional File 1 — Danish general practice. [file 1472-6963-7-46-S1.doc]

**Danish general practice**

Denmark (5.5 mio. inhabitants) is divided into 16 health administrative regions with 50,000 to 625,000 inhabitants. In each region the Public Health Insurance regulates the number of general practitioners. 98% of the inhabitants are registered (listed) with a local GP and receive tax-supported free medical care. The GPs are self-employed, but are paid by the Public Health Insurance both per capita (inhabitants on the GP’s list) and on a fee for service basis (70%/30%). The GPs serve as gatekeepers vis-à-vis the rest of the health care system (i.e. access to a hospital or specialist care implies referral by a GP) and are responsible for the care of all registered patients 24 hours a day. The GPs in a region co-operate about the out-of-hours care.

The GPs can organise their practice in several ways: A practice can be owned by one GP – a **single-handed practice**. A single-handed practice can be owned by two GPs who are working part-time – a **shared single-handed** practice. In this case the Public Health Insurance has decided that the patient list size must not exceed that of a standard single-handed practice. A **group of single-handed** GPs can share premises and staff, but not patients. A group of GPs can also own a larger practice together and serve a list of patients matching the number of GPs – a **partnership practice**. If the GPs in a partnership outnumber the list of patients in terms of patients per GP, they usually work part-time – a **part-time partnership.** In this case the Public Health Insurance also sets limits for the list size.
