# Supplementary material for: Doctor and practice characteristics associated with differences in patient evaluations of general practice
Source: BMC Health Serv Res. 2007 Apr 3;7:46. doi: 10.1186/1472-6963-7-46 (PMC1855053; doi:10.1186/1472-6963-7-46)
Supplement: Additional File 2 — The EUROPEP questionnaire. [file 1472-6963-7-46-S2.doc]

**The EUROPEP-questionnaire**

# Doctor-patient-relationship

# What is your opinion of the general practitioner and/or general practice over the last 12 months with respect to...

1. making you feel you had time during consultations?

# 2. interest in your personal situation?

# 3. making it easy for you to tell him or her about your problems?

# 4. involving you in decisions about your medical care?

5. listening to you?

6. keeping your records and data confidential?

**Medical care**

# What is your opinion of the general practitioner and/or general practice over the last 12 months with respect to...

7. quick relief of your symptoms?

8. helping you to feel well so that you can perform your normal daily activities?

9. thoroughness?

10. physical examination of you?

11. offering you services for preventing diseases?

**Information and support**

# What is your opinion of the general practitioner and/or general practice over the last 12 months with respect to...

12.explaining the purpose of tests and treatments (eg. screening, health checks, immunisations)?

13. telling you what you wanted to know about your symptoms and/or illness?

14. helping you deal with emotional problems related to your health status?

15. helping you understand the importance of following his or her advice?

**Organisation of care**

# What is your opinion of the general practitioner and/or general practice over the last 12 months with respect to...

16. knowing what he or she had done or told you during contacts?

17. preparing you for what to expect from specialist or hospital care?

**Accessibility**

# What is your opinion of the general practitioner and/or general practice over the last 12 months with respect to...

18. the helpfulness of the staff (other than doctor)?

19. getting an appointment to suit you?

20. getting through to the practice on the phone?

21. being able to speak to the general practitioner on the telephone?

22. waiting time in the waiting room?

23. providing quick services for urgent health problems?
